# Cervical disc herniation presenting with neck pain and contralateral symptoms: a case report

**DOI:** 10.1186/1752-1947-6-166

**Published:** 2012-06-28

**Authors:** Jacky T Yeung, John I Johnson, Aftab S Karim

**Affiliations:** 1 Department of Surgery, Michigan State University College of Human Medicine, 500 Perry Road, Flint, MI, 48439, USA; 2 Department of Radiology, Division of Anatomy, Michigan State University College of Human Medicine A519 East Fee Hall, 965 Fee Road, East Lansing, Michigan, 48824-1316

**Keywords:** Cervical, Contralateral symptoms, Disc herniation, Radiculopathy

## Abstract

**Introduction:**

Cervical disc herniation often results in neck and arm pain in patients as a result of direct impingement of nerve roots and associated inflammatory processes. The clinical presentation usually corresponds with the side of herniation and ipsilateral symptoms predominate the clinical picture.

**Case presentation:**

A 35-year-old Caucasian man presented to our facility with neck pain and left-sided upper and lower extremity pain. A magnetic resonance imaging scan revealed a right paramedian herniated disc at the C5 to C6 level. All other cervical levels were normal without central canal stenosis or neural foraminal stenosis. Results from magnetic reasonance imaging scans of the brain and lumbar spine were negative. An anterior cervical discectomy was performed at the C5 to C6 level, and an inter-body graft and plate were placed. Our patient had complete resolution of his neck and left arm pain.

**Conclusions:**

Anterior discectomy and fusion of the cervical spine resulted in complete resolution of our patient’s neck and left arm symptoms and improvement of his contralateral left leg pain. Cervical disc herniation may present with contralateral symptoms that are different from the current perception of this disease.

## Introduction

Cervical disc herniation results from the displacement of the nucleus pulposus of the inter-vertebral disc at the cervical level, which may result in direct compression of the spinal cord or impingement of nerve roots. Herniation of the nucleus pulposus (HNP) at the cervical level often results in radiculopathy, marked by compression and inflammation of the cervical nerve root near the neural foramen. Cervical HNP can be generally classified into four types: disc bulge, protrusion, extrusion, and sequestration [1]. Herniation in general is considered to be the result of posterolateral annular stress compounded by natural degeneration of the disc [2].

The symptoms of posterolateral cervical herniation present as ipsilateral pain in the neck, or pain radiating down the ipsilateral arm to the fingers. The pain can be dull or sharp in quality. Numbness or tingling may also replace pain as the primary presentation. Neck flexion and arm abduction over the top of the head may yield the same effect. Furthermore, decreased sensation to pain, touch, or vibration may be present in the ipsilateral arm. Although not absolute, the ipsilateral nature of the above symptoms is a hallmark of cervical herniation disrupting the nerve roots on the same side. Rarely, Brown-Séquard syndrome may develop as a result of the herniation compressing the anterior spinal cord, causing ipsilateral weakness and contralateral loss of pain and temperature at and below the affected level [3-5].

We report the case of a patient with a right-sided C5 to C6 disc herniation who presented with neck pain and left-sided pain of the upper and lower extremities. In the absence of any other objective pathology on imaging, no explanations for our patient’s contralateral symptoms were found with the exception of the right-sided herniation. Anterior cervical discectomy and fusion resulted in complete resolution of his neck and left arm pain. To the best of our knowledge, except for cases involving Brown-Séquard syndrome, a case of this nature has not been reported previously.

## Case presentation

A 35-year-old Caucasian man was evaluated at our facility for neck pain radiating to his left arm and left leg. He had numbness in his left leg and foot, which was accompanied by weakness in the left leg. His pain was intermittent and was worse when he was supine or sitting. The pain was alleviated with walking. Our patient had tried physical therapy and chiropractic care to no avail. He denied loss of bladder or bowel control. He denied any significant past medical and surgical history except for hypertension. His family history was unremarkable except for heart failure and myocardial infarction. He denied the use of alcohol, cigarettes, or recreational drugs. A general review was negative except for occasional headaches, difficulty sleeping, and tiredness. On physical examination our patient exhibited hyperreflexia and intermittent twitching in the lower extremities. He had no footdrop or saddle anesthesia. Motor strength and sensation of his upper and lower extremities were all normal.

**Figure 1:**
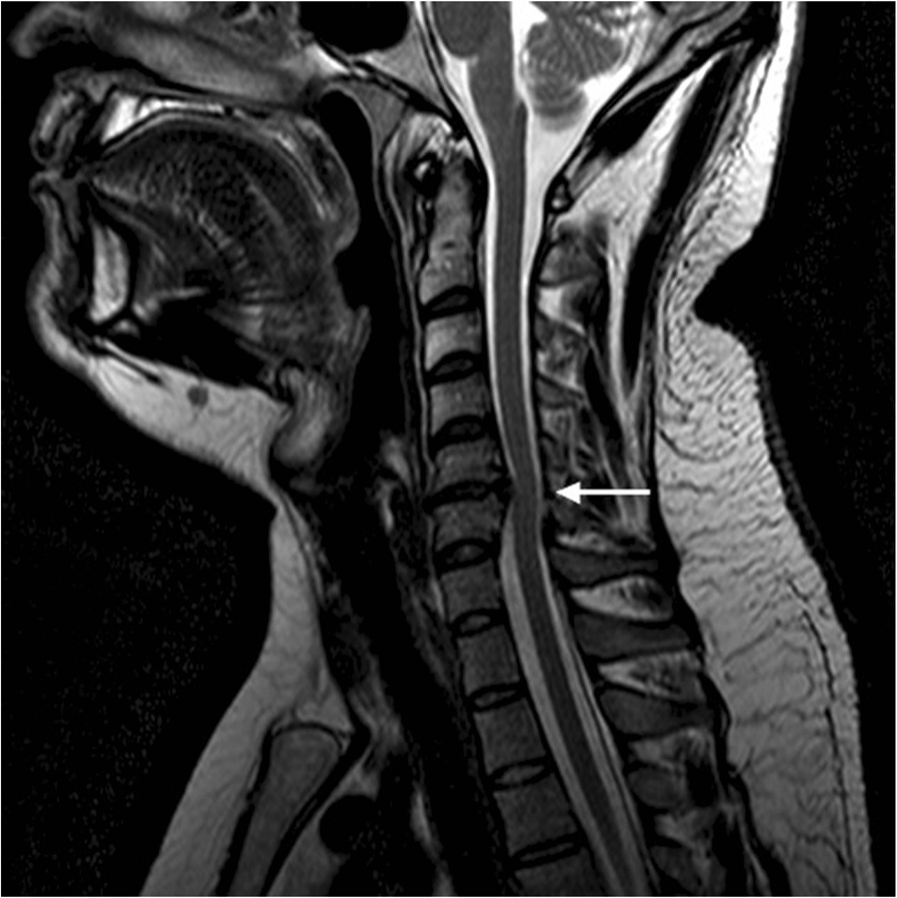
**Pre-operative magnetic resonance imaging scan.** The arrow on the sagittal T1-weighted cervical spine image shows the central canal is severely narrowed.

**Figure 2:**
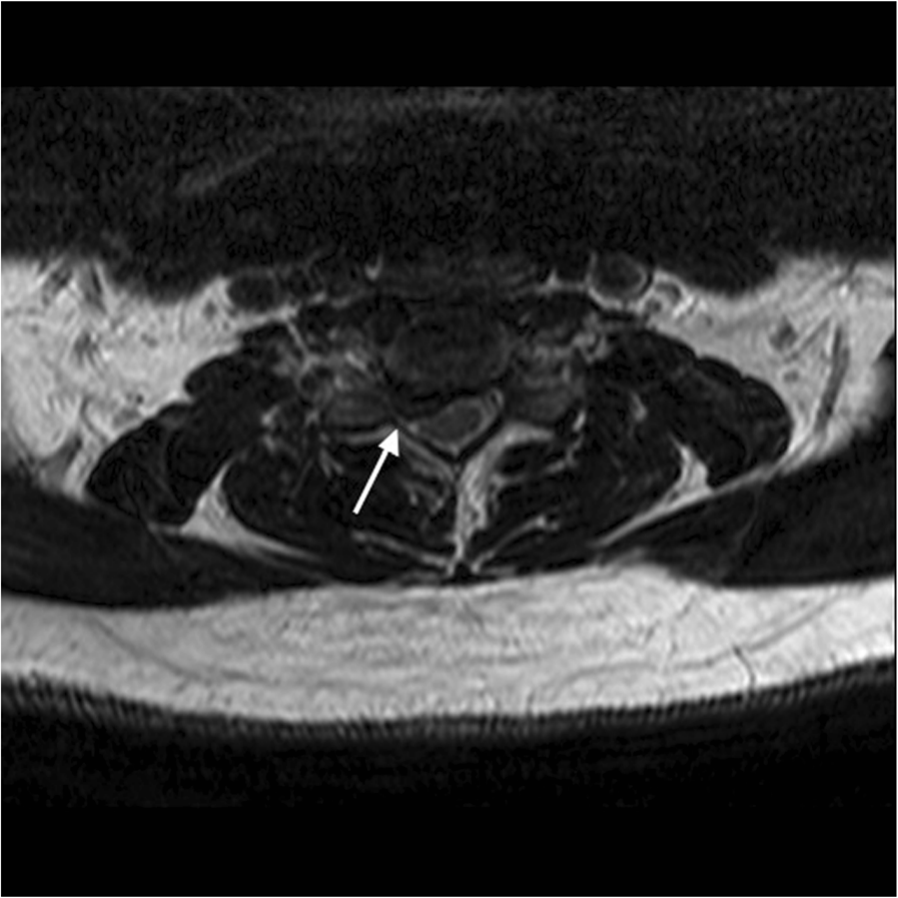
**Pre-operative magnetic resonance imaging scan.** The arrow on the axial T1-weighted cervical spine image shows the cervical cord posteriorly displaced at the level of the C5 to C6 disc space.

Routine magnetic resonance imaging (MRI) examination of the cervical spine was performed in the sagittal and axial planes using T1, T2, and short T1-inversion recovery (STIR). MRI without addition of intravenous contrast showed that there was a loss of disc height with right-sided disc herniation at the C5 to C6 level (Figures 1 and 2). All other cervical levels were normal without central canal stenosis or neural foraminal stenosis. The cervical cord was posteriorly displaced at the level of the C5 to C6 disc space. The moderately large herniation was prominent in the right paramedian aspect. The cervical cord was of normal signal. There was extension of the herniated disc in the right C6 neural foramen that compromised the exiting nerve root. The results of MRI scans of the brain and the lumbar spine were negative.

An anterior cervical discectomy was performed at the C5 to C6 level and an inter-body graft and plate were placed. The surgery was without complications.

**Figure 3:**
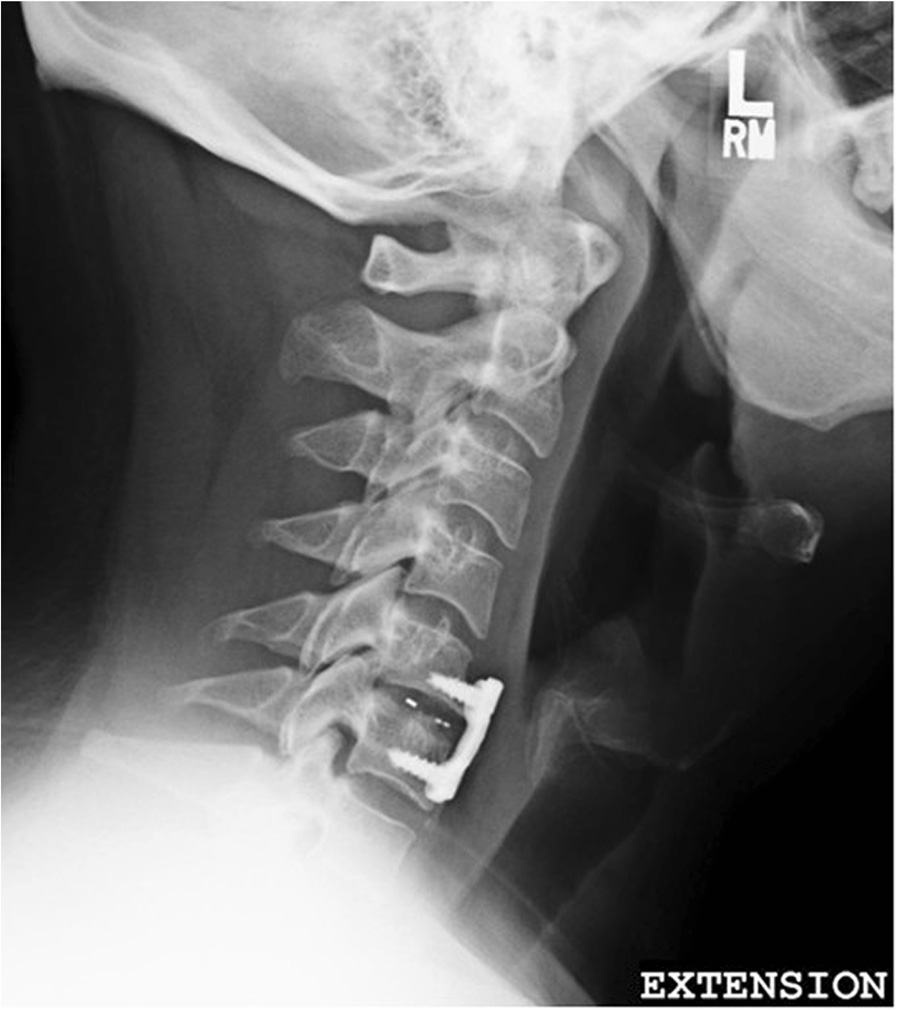
**Post-operative X-ray image.** Lateral projection showing anterior cervical fusion at the C5 to C6 level.

At 12-day follow-up our patient reported no residual pain from the surgery and resolution of left-sided symptoms. He reported resolution of all the symptoms that he had prior to his surgery, including his neck pain. X-ray imaging was completed and showed stable hardware (Figure 3).

Our patient was followed up for two years after his surgery. He was unable to attend the clinic, so instead he was questioned over the telephone about his symptoms. Our patient reported that the neck and left arm pain present before surgery were now completely resolved. He reported some residual left leg pain that was however vastly improved from before the surgery. The weakness in his left leg was also vastly improved compared to before surgery.

## Discussion

Cervical disc herniation is a condition that is seldom the result of a single traumatic event. It is a progressive disorder that typically presents as pain or weakness in the neck and arm involving ipsilateral dermatomes and myotomes that correspond to the cervical levels affected. Our patient’s presentation was atypical in that he presented with neck pain and left-sided symptoms, contralateral to his cervical lesion. It was difficult to assess whether the contralateral symptoms were the result of the right-sided cervical herniation, but the prominent herniation and compression at the C5 to C6 level, along with no imaging findings in the brain and lumbar spine, suggested no other explanations for his symptoms. Additionally, the complete resolution of our patient’s neck and left arm pain and vast improvement in his left leg symptoms by discectomy and fusion further supported the herniation to be the cause of the contralateral symptoms.

The herniation could indeed cause cervical radiculopathy by affecting the nerve roots at that spinal level, but the symptoms on the contralateral side below the level of the lesion must have been caused by the compression of the cord. There have been reports of cervical herniation resulting in Brown-Sèquard syndrome [3-5]. In those cases, ipsilateral weakness and contralateral loss of pain and temperature were the principal findings. Although different in presentation than our patient’s case, the reported cases of Brown-Séquard syndrome due to cervical herniation allowed the idea that cord compression due to cervical herniation may give rise to clinical presentations that are different to the classic radicular findings.

An explanation for our patient’s unique presentation requires consideration of the anatomy of the spinal cord. The spinothalamic tract is known for its role in pain and temperature signal transmission. When a painful stimulus is elicited, the signal enters the dorsal horn of the corresponding spinal level and synapses with the secondary neuron. The secondary neuron then crosses to the opposite side via the anterior white commissure and ascends until it terminates in the ventral posterolateral nucleus, ventral posterior oralis nucleus, ventral posterior inferior nucleus, and posterior part of the ventral medial nucleus of the thalamus [6]. The lateral spinothalamic tract is of greater clinical importance than its anterior counterpart because it transmits impulses concerned with pain. Here, we propose the possibility that this tract was disrupted by the C5 to C6 herniation and resulted in our patient’s symptomatology. In Brown-Séquard syndrome, the hemisection of the cord results in complete obliteration of the lateral spinothalamic tract, which explains the loss of pain and temperature on the contralateral side below the lesion because the fibers have already crossed by the time they reach that level. In our patient, it is possible that the compression of the cord by the herniation, which was positioned adjacent to where one would expect the lateral spinothalamic tract to run, disrupted and irritated the tract enough to generate aberrant pain transmission. Although this is not a Brown-Séquard presentation, the fiber tracts involved in the syndrome may have a role in this case. The contralateral leg pain on our patient’s left side was most likely caused by direct compression of the ipsilateral spinothalamic tract, which crosses over at the lumbrosacral levels. There was no ataxic gait in the clinical findings. The bilateral hyperreflexia and intermittent twitching of the lower limbs were non-specific signs of upper motor neuron lesions that could have been a consequence of general cord compression at the cervical level. The contralateral weakness in the left leg in this case is not easily explained anatomically and is different from traditional neuroanatomical doctrines because somehow the already-crossed contralateral corticospinal tract was affected by the ipsilateral lesion. This may be analogous to Kernohan’s notch phenomenon in the brain, where ipsilateral hemiplegia is caused by compression of the contralateral cerebral peduncle against the tentorial edge by a supratentorial pressure; the pressure generated by the herniated disc could have been transferred across the cord to affect the contralateral dorsal column and corticospinal tract on the left side, causing the feeling of numbness and weakness, respectively.

## Conclusions

In summary, our patient experienced pain in his neck radiating to his left side. No other focal neurological deficits were found, except for bilateral hyperreflexia and twitching of the lower limbs. MRI scans revealed right paramedian cervical herniation at the C5 to C6 level. Anterior discectomy and fusion of the cervical spine resulted in complete resolution of his neck and left arm symptoms and improvement of his contralateral left leg pain. It is postulated that the lateral spinothalamic tract could be involved due to cord compression by the herniated disc, resulting in predominantly contralateral symptoms. Our patient’s case may provide further insights into other atypical presentations in cervical disc herniation. Also, the current dictum that cervical disc herniation should correlate with only ipsilateral symptoms is questioned by the case presented in this manuscript.

## Consent

Written informed consent was obtained from the patient for publication of this manuscript and any accompanying images. A copy of the written consent is available for review by the Editor-in-Chief of this journal.

## Competing interests

The authors declare that they have no competing interests.

## Authors’ contributions

JY and AK analyzed and interpreted patient data regarding the cervical herniation. JY and AK were major contributors in writing the manuscript. JJ provided inputs concerning the neuroanatomy of the case. All authors read and approved the final manuscript.
